# CD24 in Melanoma: Biomarker, Innate Immune Checkpoint and Emerging Therapeutic Target

**DOI:** 10.1111/exd.70271

**Published:** 2026-05-13

**Authors:** Claudia Lasalle, Rachel C. Chang, Nicole C. Nowak, Yulu Wang, Alessio Giubellino, Kyle T. Amber, Adrian P. Mansini

**Affiliations:** ^1^ Department of Dermatology Rush University Medical Center Chicago Illinois USA; ^2^ Department of Laboratory Medicine and Pathology University of Minnesota Minneapolis Minnesota USA; ^3^ Department of Urology Rush University Medical Center Chicago Illinois USA

**Keywords:** CD24, immune evasion, innate immune checkpoint, melanoma, Siglec‐10, tumour microenvironment

## Abstract

Immune checkpoint inhibitors have transformed the treatment of advanced melanoma, yet many patients develop primary or acquired resistance. Although most work has focused on adaptive checkpoints (PD‐1 and CTLA‐4), accumulating evidence implicates innate immune suppression and stem‐like, drug‐resistant melanoma cell states. CD24, a small, heavily glycosylated glycosylphosphatidylinositol (GPI)‐anchored surface protein, sits at the intersection of these processes and is emerging as a context‐dependent biomarker and potential mediator of aggressive, therapy‐resistant melanoma states. In this review, we synthesize evidence indicating that CD24 is both a tumour‐intrinsic and tumour‐extrinsic regulator in melanoma. We summarize the structure, glycosylation and regulation of CD24, then discuss its role in melanoma, supporting phenotypic plasticity, sustaining stem‐like populations and promoting resistance to BRAF‐targeted and cytotoxic therapies through SOX2/STAT3‐linked programmes. We then examine the CD24–Siglec‐10 axis as an innate immune checkpoint that suppresses macrophage and dendritic cell function, promotes immune‐excluded ‘cold’ tumour microenvironments and may shape responses to immunotherapy among CD24+ melanoma cells. We highlight CD24 in tumour tissue, blood and extracellular vesicles as potential biomarkers of prognosis and pathway activity, and review CD24‐axis interventions, including anti‐CD24 antibodies, Siglec‐10 antagonists and CD24‐targeted CAR‐T/CAR‐NK cells, with rational combinations alongside PD‐1/CTLA‐4 blockade and MAPK‐targeted therapy. We propose that biomarker‐driven trials targeting this axis could open a new front in melanoma immunotherapy.

**Abbreviations**
ABC: ATP‐binding cassette
ADC: antibody–drug conjugate
ADCC: antibody‐dependent cellular cytotoxicity
ADCP: antibody‐dependent cellular phagocytosis
AKT: Protein kinase B
ARF: ADP‐ribosylation factor
Bcl‐2: B‐cell lymphoma 2
CAR: Chimeric antigen receptor
CAR‐NK cells: Chimeric antigen receptor natural killer cells
CAR‐T: Chimeric antigen receptor T‐cell
CD24: cluster of differentiation 24
CD271: cluster of differentiation 271
CRS: cytokine release syndrome
CSC: cancer stem cells
CTLA‐4: cytotoxic lymphocyte antigen 4
DAMPs: damage‐associated molecular pattern
DCs: dendritic cells
EMT: epithelial‐mesenchymal transition
EV: extracellular vesicle
GPI: glycosylphosphatidylinositol
HMGB1: high mobility group box 1
HSA: heat‐stable antigen
ICB: immune checkpoint blockade
IHC: immunohistochemistry
ITIM: immunoreceptor tyrosine‐based inhibitory motif
mAb: monoclonal antibody
MAPK: mitogen‐activated protein kinase
MDM2: mouse double minute 2 homologue
MDSCs: myeloid‐derived suppressor cells
MEK: MAPK/ERK kinase
MRD: minimal residual disease
NK: natural killer cell
NKG2D: natural killer group 2
NPM: nucleophosmin
Oct4: Octamer‐binding transcription factor 4
ORR: objective response rate
OS: overall survival
PAMPs: pathogen‐associated molecular pattern
PD‐1: programmed cell death protein 1
PD‐1/PD‐L1: programmed cell death‐1/programmed death‐ligand 1
PD‐L1: programmed death‐ligand 1
PFS: progression‐free survival
PI3K: phosphoinositide 3‐kinase
PK: pharmacokinetics
SHP‐1/2: Src homology region 2 domain‐containing phosphatase‐1/2
shRNA: short hairpin RNA
Siglec: sialic acid‐binding immunoglobulin‐like lectin
SIRPα: signal‐regulatory protein alpha
SOX2: SRY‐box 2
SOX4: SRY‐box 4
STAT3: signal transducer and activator of transcription 3
TAMs: tumour‐associated macrophages
TGF‐β: transforming growth factor‐beta
TME: tumour microenvironment
TRAMP: transgenic adenocarcinoma of mouse prostate

## 1 Introduction

Melanoma is a highly aggressive skin cancer with an increasing incidence worldwide [1]. Immune checkpoint blockade (ICB) targeting programmed cell death protein 1 (PD‐1) and cytotoxic T‐lymphocyte antigen 4 (CTLA‐4) has transformed treatment, leading to durable remissions in a subset of patients [2, 3]. However, 40%–60% of patients exhibit primary resistance, and many others ultimately develop acquired resistance [4, 5]. Although most mechanistic work has focused on adaptive immune checkpoints, increasing evidence indicates that innate immune suppression within the tumour microenvironment (TME), together with tumour cell plasticity and therapy‐resistance states, represents a major barrier to effective antitumor immunity.

CD24 is a small, heavily glycosylated cell‐surface protein attached to the outer leaflet of the plasma membrane through a glycosylphosphatidylinositol (GPI) anchor [6, 7, 8]. Rather than functioning as a classical transmembrane receptor with its own intracellular signalling domain, CD24 is better understood as a membrane‐organising scaffold and co‐receptor that shapes ligand interactions, membrane microdomains and downstream signalling through associated protein complexes [8, 9, 10]. In cancer, this biology has been linked to phenotypic plasticity, therapy resistance and immune evasion, although the strength and tumour‐type specificity of these associations vary substantially across studies [11, 12, 13, 14, 15, 16, 17].

One of the most important immune functions of CD24 is mediated through its interaction with sialic acid‐binding immunoglobulin‐like lectins (Siglecs), a family of inhibitory receptors expressed predominantly on myeloid cells. In human tumours, CD24 can engage Siglec‐10 to transmit an inhibitory ‘don't eat me’ signal that restrains macrophage phagocytosis and broader myeloid activation, positioning the CD24–Siglec‐10 axis as an emerging innate immune checkpoint [16].

A key translational consideration is species‐specific nomenclature and biology within the CD24–Siglec axis. In human systems, CD24 interacts with Siglec‐10 on myeloid cells, whereas murine studies commonly model the corresponding pathway through Siglec‐G [10]. Because these molecules are related but not identical, mechanistic and therapeutic conclusions from mouse models should be interpreted as supportive rather than directly interchangeable with human melanoma biology. Accordingly, humanised systems may be needed to directly validate CD24–Siglec‐10 biology and therapeutic targeting in preclinical melanoma studies. In addition, the melanoma literature remains uneven: some observations are supported by melanoma patient samples or melanoma‐specific functional studies, whereas others are extrapolated from broader solid‐tumour or immunology literature [16, 18]. Accordingly, throughout this review, we distinguish melanoma‐supported evidence from cross‐tumour hypotheses, particularly when discussing tumour‐intrinsic mechanisms, biomarker claims and therapeutic prioritization.

This review summarizes current understanding of CD24 in melanoma, including its molecular biology, tumour‐intrinsic and tumour‐extrinsic roles and clinical importance. We also examine emerging therapeutic strategies that target the CD24–Siglec‐10 axis and discuss how these approaches may be integrated with existing melanoma therapies.

## 2 CD24 Biology and Signalling

### 2.1 Structure and Glycosylation

**FIGURE 1:**
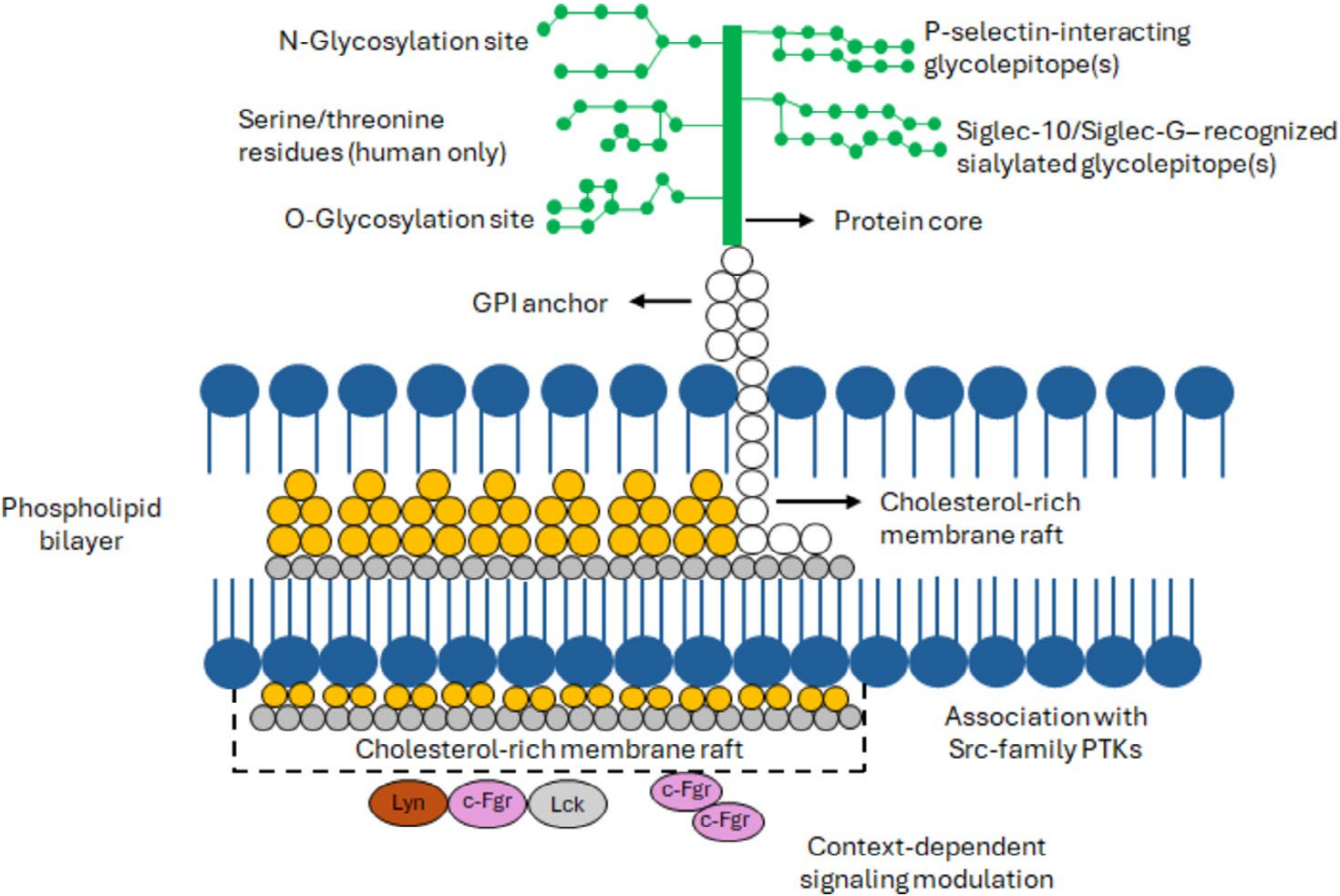
Structure and glycosylation of CD24. CD24 is a small, heavily glycosylated GPI‐anchored cell‐surface protein with a compact protein core, multiple N‐ and O‐glycosylation sites and context‐dependent ligand‐binding properties. Its localization in cholesterol‐rich membrane rafts supports interactions with associated membrane proteins and signalling complexes, consistent with a scaffold/co‐receptor role rather than intrinsic intracellular signalling activity.

CD24 is a small, heavily glycosylated cell‐surface protein attached to the outer leaflet of the plasma membrane via a glycosylphosphatidylinositol (GPI) anchor [6, 7]. In humans, the mature protein is produced from a short precursor (82 amino acids) after the removal of the N‐terminal signal peptide and the C‐terminal GPI‐attachment sequence, along with extensive post‐translational glycosylation. This results in a highly heterogeneous glycoprotein, ranging from 20 to 70 kDa [6]. CD24 is characterized by a compact protein core decorated with multiple N‐linked and O‐linked glycans, which are crucial for ligand recognition and immunomodulatory activity (Figure 1).

This extensive glycosylation creates a versatile extracellular interface and is likely a major determinant of context‐dependent CD24 function [6, 19]. Because CD24 is GPI‐anchored and enriched in lipid rafts, it is best viewed not as a classical signalling receptor but as a membrane‐organising scaffold and co‐receptor that helps shape ligand accessibility, membrane microdomains and signalling context [6, 20, 21]. In cancer, altered glycosyltransferase activity and broader glycocalyx remodelling may influence CD24 abundance, glyco‐epitope presentation and binding properties [19]. This structural heterogeneity may also contribute to the variable performance of CD24 as a biomarker and therapeutic target across tumour types [20].

### 2.2 Ligands and Associated Downstream Pathways

CD24 functions as an innate immune ‘danger’ modulator through interactions with Siglec family receptors on myeloid cells. In humans, CD24 binds Siglec‐10, whereas murine studies commonly model the corresponding pathway through Siglec‐G [8, 22]. This sialic acid‐dependent axis helps discriminate endogenous damage‐associated molecular patterns (DAMPs) from pathogen‐associated molecular patterns (PAMPs), thereby restraining excessive sterile inflammation [8, 22]. Loss of sialylation disrupts CD24–Siglec binding and amplifies DAMP‐driven cytokine responses, whereas soluble or recombinant CD24‐Fc can partially restore this regulatory axis and has shown anti‐inflammatory activity in preclinical sepsis models and in clinical studies of hyperinflammatory states [23, 24]. Collectively, these findings establish CD24 as a context‐dependent regulator of the threshold for innate immune activation rather than merely an adhesion‐associated surface marker.

Beyond this immunoregulatory role, CD24 has been associated with signalling‐active membrane microdomains in cancer cells. Through its localization in lipid rafts and its association with other membrane proteins, CD24 may facilitate the assembly of signalling‐competent complexes involving Src‐family kinases and related effectors [20, 21, 25, 26]. Across cancer models, CD24 expression has been linked to pathways such as Src/STAT3, PI3K/AKT and MAPK, together with phenotypes including proliferation, survival, migration, adhesion and stress adaptation [11, 20, 25, 27, 28]. However, much of this mechanistic evidence derives from non‐melanoma systems; therefore, these pathway assignments should be interpreted in melanoma as supported hypotheses unless validated by melanoma‐specific perturbation studies [29, 30]. In this context, tumour‐cell CD24 is more appropriately viewed as a membrane‐associated organizer of signalling context and ligand availability than as a receptor with intrinsic intracellular signalling capacity.

CD24 may also intersect with canonical tumour‐suppressor pathways in selected cancer contexts. In prostate cancer, including TRAMP (transgenic adenocarcinoma of mouse prostate) models, intracellular CD24 was shown to competitively disrupt the ARF–nucleophosmin (NPM) interaction, resulting in reduced ARF levels, increased MDM2 and decreased p53 and p21/CDKN1A expression [31]. In the same study, targeted Cd24 deletion and shRNA‐mediated CD24 silencing retarded prostate cancer growth, progression and metastasis, supporting a functional role for CD24 in attenuation of the ARF–MDM2–p53 axis. Whether this mechanism also operates in melanoma remains unknown and should currently be regarded as a cross‐tumour hypothesis rather than established melanoma biology.

### 2.3 Regulation and Expression

CD24 is encoded by a single gene, but alternative splicing may generate isoforms with distinct effects on membrane association, subcellular localization and interaction context [6]. Although isoform‐specific CD24 biology is increasingly recognized in other cancers, melanoma‐specific data on CD24 isoform expression and function remain limited [32]. This gap is likely to be translationally relevant, as isoform and glycoform diversity may influence antibody recognition, biomarker interpretation and therapeutic targetability [19, 33]. Accordingly, CD24 will likely need to be interpreted in a context‐aware manner rather than through single‐epitope readouts alone.

CD24 expression is tightly regulated according to tissue type and differentiation stage [21]. In mouse haematopoiesis, the heat‐stable antigen (HSA; the precursor to CD24) is predominantly expressed on immature progenitors and declines with terminal differentiation [9, 21]. Consistent with this pattern, B‐cell precursors express high levels of CD24, whereas plasma cells largely lack it [9, 21]. CD24 is also present on subsets of T lymphocytes, monocytes, granulocytes and several non‐haematopoietic lineages, including epithelial, neural and muscle cells [6, 20, 21]. Depending on cellular context, CD24 may function as a co‐stimulatory or broader immunoregulatory molecule [9].

In cancer, this regulatory programme can be co‐opted. CD24 overexpression has been associated with increased migration, invasion and proliferation across multiple tumour types [11, 20, 27]. Meta‐analytic data further link CD24 positivity with nodal metastasis, advanced clinical stage and worse survival [27]. Mechanistically, CD24 can facilitate metastatic dissemination through interactions with P‐ and E‐selectin, promoting endothelial rolling and extravasation [34, 35]. Isoform‐specific effects have also been described, with CD24A driving stronger proliferative and invasive phenotypes than CD24B in hepatocellular carcinoma models [36].

Local microenvironmental cues and lineage‐specific transcriptional programmes also shape CD24 expression [6, 20, 21]. In urothelial carcinoma, androgen receptor signalling increased CD24 promoter activity, whereas in breast cancer, oestrogen receptor‐α suppressed CD24 through oestrogen‐responsive elements in the CD24 promoter [37]. Although androgen receptor signalling is relevant to melanoma biology, a direct melanoma‐specific AR–CD24 regulatory link has not yet been established [38, 39].

In melanoma, baseline CD24 expression is often low or heterogeneous, but therapy and cell‐state transitions can increase its expression [7, 32, 39, 40]. BRAF inhibition has been reported to induce CD24 through a SOX2‐dependent adaptive programme, thereby promoting drug‐resistant phenotypes [39]. TGF‐β/SOX4 signalling may further converge on SOX2, and STAT3 has also been implicated in pathways leading to CD24 induction [29, 39, 41]. However, SOX2 function is highly context dependent in melanoma, underscoring that CD24 regulation should be interpreted in light of cell state, transcriptional network context, genetic background and therapeutic pressure [39, 40, 42, 43].

## 3 CD24 Expression in Melanoma

### 3.1 Tumour Tissue Expression and Clinicopathologic Correlations

CD24 is expressed in a subset of primary and metastatic melanomas; however, its prevalence and clinicopathologic significance appear to be context dependent [7, 32]. Immunohistochemical studies have generally shown low to moderate membranous and/or cytoplasmic CD24 staining in melanoma cells, with higher levels reported in more advanced or metastatic lesions than in benign nevi or in situ disease [7, 32]. Across multiple solid tumours, cytoplasmic and membranous CD24 expression has been associated with higher tumour grade, metastatic spread and worse survival [27]. In cutaneous melanoma, the available literature points in a similar direction, but the evidence base remains relatively limited and should be interpreted cautiously as supportive rather than definitive [7, 32].

Importantly, CD24 expression alone does not define a specific melanoma lineage or histologic subtype. Instead, tumour‐cell CD24 is better viewed as a context‐dependent marker of aggressive cell states, including phenotypes linked to migration, invasion, stem‐like behaviour or therapy resistance [30, 32, 39, 40]. This interpretation is supported by experimental melanoma models, in which CD24‐high cells exhibit enhanced migration, sphere‐forming capacity and tumour‐initiating potential relative to CD24‐low cells [30, 32]. Accordingly, when discussing prognosis, it is more precise to frame CD24 as a candidate biomarker enriched in adverse melanoma states than as a universally validated stand‐alone prognostic marker.

### 3.2 Phenotypic and Subtype‐Specific Expression

Analyses across melanoma subtypes, including superficial spreading, nodular and acral lentiginous melanoma, have not identified a robust subtype‐specific pattern of CD24 expression [7, 32]. Rather, CD24 varies both within and between tumours, often co‐occurring with broader programmes of stemness, invasion and plasticity rather than with a fixed histopathologic identity [29, 30, 32, 43]. This intratumoral heterogeneity is consistent with the broader concept that melanoma cells transition between proliferative, invasive, therapy‐resistant and dedifferentiated states under microenvironmental and therapeutic pressure [29, 30, 40, 43].

In this setting, CD24 should not be interpreted as a simple classifier, but rather as one component of a dynamic phenotype. Consistent with this view, studies in other tumour contexts have linked CD24 expression to activation of oncogenic signalling pathways, including Notch1, MAPK and PI3K/AKT [6, 28, 44], further supporting its role as a facilitator of aggressive traits rather than a simple classification marker. Melanoma models support this view by linking CD24 expression to aggressive traits and by showing that CD24‐high states can overlap with stem‐like properties and enhanced adaptive capacity [30, 32, 39, 40]. However, several mechanistic associations often cited in the broader CD24 literature derive from non‐melanoma systems; therefore, pathway‐level interpretations should be restricted to melanoma‐supported evidence where available and otherwise presented as cross‐tumour hypotheses [10, 20, 25, 28]. From a translational standpoint, these features support evaluating CD24 in single‐cell, spatial or multimarker contexts, ideally alongside additional state descriptors such as CD271, SOX2/STAT3‐associated programmes, epithelial‐mesenchymal transition (EMT)‐related features and metabolic adaptation markers, rather than using CD24 alone as a categorical readout [29, 30, 39, 40, 43].

### 3.3 Circulating and Extracellular Vesicle‐Associated CD24

Beyond tissue expression, CD24 can also be found in circulation, including on EVs such as exosomes [20, 45, 46]. Exosomes are small EVs released by various cell types that carry proteins, lipids, metabolites and nucleic acids, supporting communication between nearby and distant cells [45, 46]. This vesicle secretion is especially active in proliferating cells, including cancer cells and tumour‐derived exosomes can influence immune suppression or activation, metastatic spread and therapy responses. Consistent with its enrichment in lipid rafts, CD24 is efficiently incorporated into EV membranes, making EV‐associated CD24 a plausible liquid‐biopsy biomarker [20, 26, 45, 46]. In melanoma, CD24‐positive EVs have been detected in patient serum, supporting the feasibility of measuring this axis outside of tumour tissue [20, 45, 46].

At the same time, the interpretation of circulating CD24 requires caution. Plasma EV populations in patients with melanoma are often dominated by vesicles derived from blood cells, and tumour‐derived EV may be underrepresented [45]. As a result, detection of circulating or EV‐associated CD24 cannot automatically be assumed to reflect tumour‐cell CD24. Instead, the cellular origin of the signal must be considered explicitly, particularly because CD24 may also derive from immune cells or other non‐tumour compartments [21, 47].

Despite these limitations, early EV‐profiling work suggests that melanoma‐derived EV can carry CD24, along with additional immunomodulatory or pro‐metastatic cargo, and that composite EV signatures may distinguish melanoma patients from healthy controls and correlate with disease burden or stage [45, 46]. In the context of the CD24–Siglec‐10 axis, vesicle‐bound CD24 is particularly interesting because it could extend innate immune suppression beyond the immediate tumour cell surface by engaging Siglec‐10‐expressing myeloid cells at local or distant sites [8, 10, 16]. If validated in larger and better‐annotated cohorts, circulating or EV‐associated CD24 may therefore serve as a minimally invasive biomarker of CD24‐axis activity and a pharmacodynamic readout for CD24‐directed therapies [20, 45, 46, 48].

## 4 Tumour‐Intrinsic Roles of CD24 in Melanoma

Beyond its role in innate immune evasion, CD24 is also associated with tumour‐intrinsic programmes in melanoma that relate to stem‐like behaviour, phenotypic plasticity, therapy resistance and stress survival [30, 32, 39, 40, 49]. However, the strength of the available evidence varies by context. In melanoma, CD24 is best viewed at present as a marker of aggressive and therapy‐resistant cell states, with selected functional studies supporting a contributory role in specific settings rather than establishing CD24 as a universal melanoma dependency [30, 32, 39, 49]. This distinction is important because some mechanistic interpretations derive from perturbation experiments in melanoma, whereas others reflect broader conceptual links drawn from cancer stem cell biology, stress adaptation and cross‐tumour CD24 literature [15, 20, 28, 40].

### 4.1 CD24 as a Marker of Melanoma Stem‐Like States

Cancer stem cells are considered important contributors to melanoma initiation, persistence and recurrence [40, 50]. Within this framework, CD24 has emerged as a marker of melanoma subpopulations enriched for tumour‐initiating and stem‐like properties. Tang et al. provided key melanoma‐specific functional evidence by showing that CD24‐positive melanoma cells displayed greater clonogenicity, sphere‐forming efficiency, migration and tumour‐initiating capacity than CD24‐negative cells in immunodeficient mice [32]. These findings support the use of CD24 as a marker of melanoma cells with enhanced tumourigenic potential.

At the same time, CD24‐positive states in melanoma appear to be plastic rather than fixed. Reversible shifts between CD24‐positive and CD24‐negative populations have been described in association with intratumoral heterogeneity, suggesting that CD24 marks a dynamic state that may expand or contract in response to environmental or therapeutic pressure [32]. This interpretation is consistent with broader models of melanoma plasticity in which cells transition between proliferative, invasive, stem‐like and drug‐resistant phenotypes [39, 40, 43, 50].

Mechanistically, melanoma studies support an association between CD24 expression and SOX2/STAT3‐linked adaptive programmes [29, 39]. BRAF‐targeted therapy can induce SOX2, which in turn promotes CD24 expression, and interference with this axis restores drug sensitivity in melanoma models [39]. These data support a model in which CD24 participates in, or is enriched within, a self‐reinforcing adaptive state linked to stemness and therapy resistance. CD24‐positive melanoma cells have also been associated with higher expression of stemness‐related transcription factors and pathways, such as Notch1, relevant to self‐renewal and invasion [30, 32, 44]. However, these observations should be interpreted as evidence of state enrichment and pathway association rather than proof that CD24 alone directly drives each downstream feature.

Metabolically, CD24‐positive melanoma states have been linked to retained metabolic activity and broader stress adaptability, features that could support survival under nutrient limitation or treatment pressure [49]. Taken together, current evidence supports CD24 as a useful marker of melanoma stem‐like states and suggests that it may contribute functionally to their maintenance in selected contexts, while stopping short of establishing CD24 as a universally dominant tumour‐intrinsic driver.

### 4.2 The CD24^+^ CD271^+^ Double‐Positive Stem‐Like Population

CD271 (NGFR) is a well‐established marker of neural crest–like melanoma cells and has been implicated in tumour initiation, migratory behaviour and therapy resistance [50, 51]. Recent work identified a rare but functionally important CD24^+^CD271^+^ melanoma population with hybrid characteristics not fully captured by either single‐marker subset [30]. This double‐positive population, identified in approximately 10% of melanoma samples, showed enhanced sphere‐forming ability, greater lineage plasticity and increased capacity to adapt to environmental stress [30].

Functionally, CD24^+^CD271^+^ melanoma cells exhibited increased motility and invasiveness, together with enrichment of migration‐related programs [30]. This observation is conceptually consistent with prior work linking CD271 to melanoma migration and metastatic behaviour [51]. In addition, the double‐positive compartment showed stronger resistance to targeted and cytotoxic therapies, suggesting that CD24 and CD271 together may identify a particularly adaptable stem‐like state rather than a static phenotypic class [30]. EMT‐related and stress‐adaptive programmes have been implicated in this context, although such pathway‐level inferences should be interpreted cautiously when direct perturbation evidence remains limited [30, 40].

Overall, the CD24^+^CD271^+^ population is best framed as a melanoma substate enriched for stem‐like, invasive and therapy‐resistant properties. It is an important example of why CD24 should be interpreted in a multimarker context rather than as a stand‐alone classifier of melanoma biology.

### 4.3 CD24 and Adaptive Therapy Resistance

Adaptive resistance to BRAF inhibition remains a major challenge in melanoma, and the strongest melanoma‐specific functional evidence supporting a tumour‐intrinsic role for CD24 comes from this setting [39, 40]. Hüser et al. showed that BRAF inhibition induces SOX2, which in turn upregulates CD24 and is associated with a slow‐cycling, stem‐like, drug‐resistant phenotype [39]. Importantly, genetic silencing of CD24 or pharmacologic inhibition of Src/STAT3‐linked signalling restored sensitivity to BRAF‐targeted therapy, indicating that CD24 is not merely a passive bystander in this adaptive programme [39].

Beyond targeted therapy, broader cancer literature links CD24+ states to increased expression of ATP‐binding cassette (ABC) transporters, anti‐apoptotic regulators such as Bcl‐2, enhanced DNA damage tolerance and metabolic reprogramming [14, 28]. These observations provide plausible mechanisms by which CD24‐enriched melanoma cells may develop cross‐resistance to multiple therapies, although direct melanoma‐specific evidence is stronger for adaptive targeted‐therapy resistance than for chemotherapy resistance per se [28, 39, 40]. Therefore, it is more accurate to state that CD24 is associated with, and in some melanoma models contributes to, survival programmes that support therapy resistance.

More broadly, these findings support a model in which CD24 marks an integrated stress‐adaptive state linking stemness, metabolic flexibility and drug resistance. This interpretation also helps connect tumour‐intrinsic and tumour‐extrinsic CD24 biology, because therapy‐resistant CD24‐high melanoma states may be especially well positioned to coexist with innate immune evasion programmes discussed in the next section.

### 4.4 CD24 Marks Anastasis‐Prone Melanoma Subpopulations

Recent work has expanded the relevance of CD24 beyond stemness and drug resistance by identifying a melanoma subpopulation in which cell‐surface CD24 enriches cells that have initiated apoptosis yet retain the capacity to recover, a phenomenon termed anastasis [49]. In B16‐F10 and YUMM5.2 models, CD24 surface expression was increased in a non‐adherent FSC^low^SSC^high^ compartment that might otherwise be excluded by conventional flow‐cytometric gating strategies [49]. Although many of these cells displayed apoptotic characteristics, the CD24‐enriched fraction retained metabolic activity and proliferative potential, including anchorage‐independent growth, consistent with apoptosis reversal (anastasis) [49].

These findings suggest that CD24 may mark detached, stress‐exposed melanoma cells capable of persistence and regrowth after apparent commitment to cell death [49]. At present, this should be interpreted as an emerging melanoma‐specific observation rather than a fully established general mechanism. Nevertheless, it broadens the conceptual framework of CD24 biology by linking CD24 not only to stem‐like and therapy‐resistant states, but also to anastasis‐prone subpopulations with potential relevance to minimal residual disease, recurrence and dissemination [49].

Notably, PD‐L1 (CD274) was detectable in this compartment, raising the possibility that anastasis‐prone CD24‐enriched states may intersect with adaptive immune checkpoint programmes [49]. This observation provides additional rationale for considering CD24 within a broader state‐based model of melanoma persistence that spans both tumour‐cell plasticity and immune evasion.

## 5 Tumour‐Extrinsic/Immune Roles: The CD24–Siglec‐10 Axis

**FIGURE 2:**
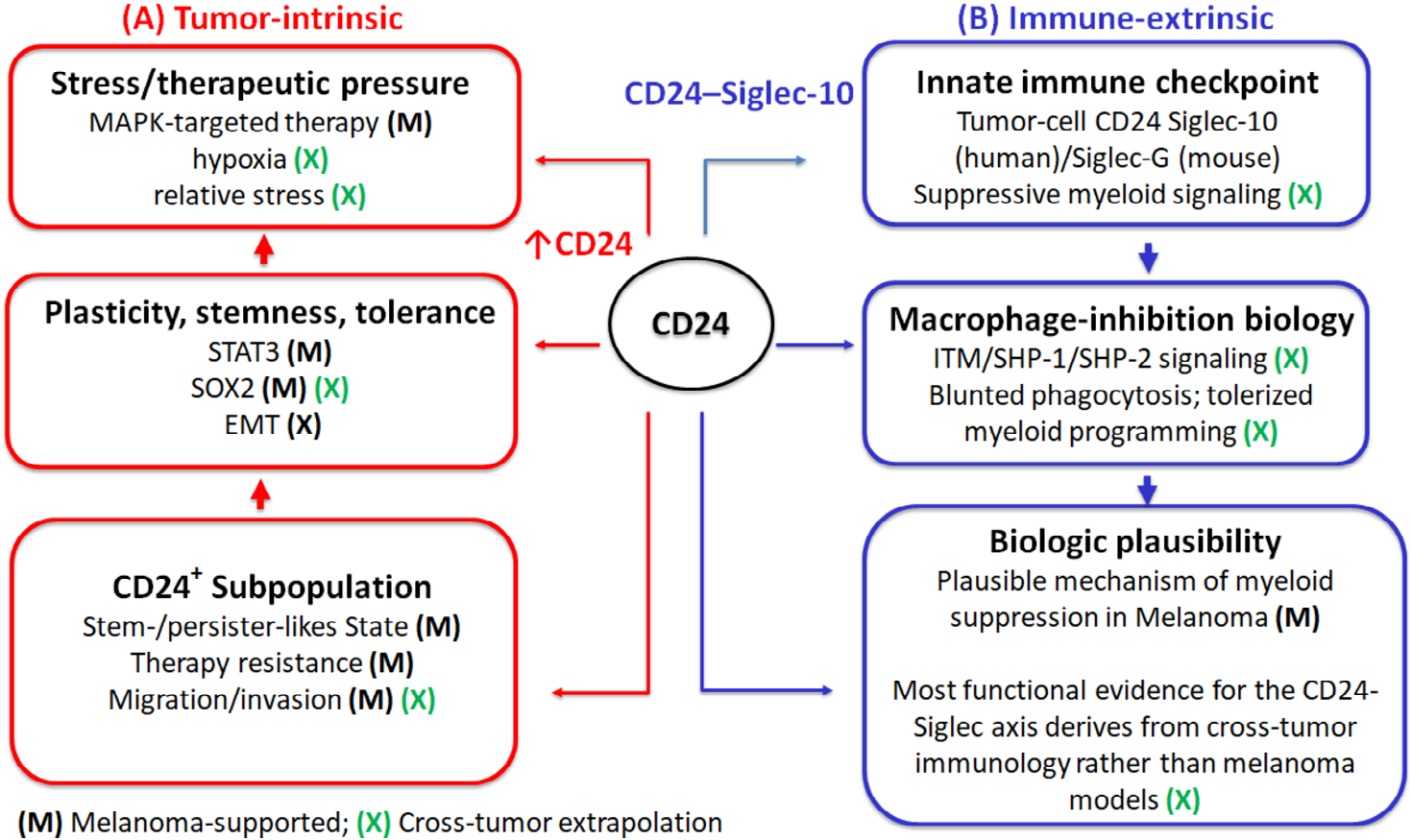
CD24 links tumour‐intrinsic melanoma plasticity with tumour‐extrinsic innate immune suppression. (A) Tumour‐intrinsic context: Stress/therapeutic pressure (e.g., MAPK‐targeted therapy) is associated with CD24 enrichment in plasticity/stemness/resistance‐linked melanoma states, including CD24‐high subpopulations. CD24 should be interpreted as a context‐dependent state marker; ‘CD24‐axis activity’ is best operationalized using context‐aware readouts (multimarker plus spatial/orthogonal assays) rather than single‐epitope measurements. (B) Tumour‐extrinsic context: Tumour‐cell CD24 can engage Siglec‐10 in humans (or Siglec‐G in mice) on myeloid cells, promoting inhibitory signalling and suppressed phagocytosis/antigen‐handling functions that support tolerized myeloid programming. (M) denotes elements supported by melanoma patient data and/or melanoma models; (X) denotes elements primarily supported by cross‐tumour literature and presented as translational extrapolations for melanoma.

While CD24 is linked to tumour‐intrinsic melanoma states, it also has important tumour‐extrinsic functions within the microenvironment through regulation of myeloid immunity. In this context, the CD24–Siglec axis may help melanoma cells evade innate immune clearance by restraining phagocytic and inflammatory myeloid programmes [8, 10, 16, 17, 52]. Human data primarily implicate Siglec‐10, whereas murine studies model the pathway through Siglec‐G; these findings should therefore be viewed as mechanistically supportive rather than directly equivalent [8, 10, 22]. Together, they support the CD24–Siglec axis as a plausible innate immune checkpoint that complements the adaptive checkpoint landscape in melanoma [8, 10, 16, 17, 53]. The tumour‐intrinsic and immune‐extrinsic functions of CD24 in melanoma are summarized in Figure 2.

A useful comparison is the CD47–SIRPα axis [54]. Both pathways deliver inhibitory ‘don't eat me’ signals to phagocytes and converge on suppression of macrophage‐mediated clearance, but they differ in ligand biology, receptor distribution and contextual regulation [10, 16, 54, 55]. CD47 is broadly expressed and signals primarily through SIRPα, whereas CD24 is a heavily glycosylated GPI‐anchored molecule whose immunoregulatory effects depend on sialylation status, glyco‐epitope context and engagement of Siglec receptors on myeloid cells [8, 10, 16, 19, 21, 55]. In melanoma, this distinction may be particularly relevant because CD24 may link tumour‐cell plasticity with innate immune suppression in ways not fully captured by the CD47 literature alone.

### 5.1 Effects on Macrophages and Myeloid‐Derived Suppressor Cells

The strongest evidence supporting the CD24–Siglec axis as an innate immune checkpoint comes from macrophage‐focused studies, in which Siglec‐10/G engagement suppresses pro‐phagocytic signalling and restrains inflammatory activation through ITIM‐dependent recruitment of SHP phosphatases [8, 10, 16, 22]. Functionally, this blunts macrophage engulfment and creates a checkpoint conceptually analogous to CD47–SIRPα [10, 15, 16, 54, 55].

Barkal et al. provided seminal functional evidence that disrupting CD24–Siglec‐10 signalling increases macrophage phagocytosis of tumour cells and constrains tumour growth in xenograft models [16]. Subsequent reviews and preclinical studies further suggest that this pathway operates broadly within the tumour myeloid compartment, as both M1‐like and M2‐like tumour‐associated macrophages can express Siglec‐10, while tumour‐derived CD24 has been linked to polarization toward a more tumour‐supportive, M2‐like state characterized by reduced phagocytic activity, increased anti‐inflammatory mediator expression and diminished support for cytotoxic T‐cell responses [10, 17, 52, 55]. However, these data were not generated in melanoma‐specific models. In melanoma, CD24–Siglec‐10 is therefore best viewed as a biologically plausible innate checkpoint supported by broader tumour immunology literature rather than as a pathway functionally validated to the same extent as in other tumour types.

Beyond macrophages, the CD24 axis may also influence other suppressive myeloid populations. In murine systems, CD24a loss has been associated with enhanced macrophage‐ and CD8^+^ T cell‐mediated antitumor immunity and with remodelling of the myeloid compartment toward a less suppressive phenotype [52]. These observations support the idea that the pathway can shape myeloid‐derived suppressor cell (MDSCs) behaviour, antigen handling and T‐cell priming indirectly through myeloid reprogramming [10, 52]. Again, this should be interpreted as supportive cross‐tumour evidence rather than melanoma‐specific proof.

In melanoma, the available evidence is more circumstantial but still relevant. CD24 is expressed in a subset of melanomas and is associated with more aggressive clinicopathologic features, while broader melanoma biomarker analyses support the relevance of myeloid‐mediated immune suppression in this disease [7]. Together with the known biology of tumour‐infiltrating myeloid cells in melanoma, these data support prioritizing the CD24–Siglec‐10 axis as a plausible mechanism of impaired phagocytic clearance and myeloid immunosuppression in melanoma, while acknowledging that direct melanoma‐specific functional perturbation studies remain limited [16].

Importantly, CD24‐associated melanoma cell states may intersect with adaptive immune evasion programmes. As discussed in Section 4.4, PD‐L1 (CD274) was detectable in a CD24‐enriched anastasis‐prone melanoma compartment, suggesting that CD24‐high states may coexist with adaptive checkpoint programmes and providing a rationale for combinatorial strategies that pair innate and adaptive checkpoint blockade [49, 56].

### 5.2 Dendritic Cells, NK Cells and Cross‐Talk With Adaptive Immunity

The immunologic impact of CD24 likely extends beyond macrophages. In principle, CD24–Siglec‐10 signalling may suppress dendritic cell (DC) maturation, antigen uptake and antigen presentation, thereby limiting effective T‐cell priming and favouring a non‐inflamed or immune‐excluded tumour microenvironment [10]. This framework is biologically coherent and attractive for melanoma, a disease in which response to immunotherapy is tightly linked to efficient antigen presentation and productive T‐cell recruitment [53]. However, the evidence supporting these DC‐centred effects comes largely from broader innate immune literature and cross‐tumour interpretation rather than melanoma‐specific functional studies [10].

CD24 may also influence natural killer (NK) cell function through both Siglec‐dependent and Siglec‐independent mechanisms. In addition to myeloid checkpoint signalling, CD24 has been reported to interact with NKG2D and thereby dampen NK‐cell activation in preclinical settings [55]. More broadly, anti‐CD24 therapeutic strategies have been associated with reversal of macrophage and NK‐cell suppression and with enhancement of CD8^+^ T‐cell responses, further supporting a model in which CD24 blockade can influence both innate and adaptive antitumor immunity [52, 55]. These findings are important translationally, but they should again be described as preclinical and predominantly cross‐tumour rather than melanoma‐specific evidence.

Taken together, the most defensible melanoma‐centred conclusion is that the CD24 axis may contribute to an immune‐cold or immune‐excluded phenotype by simultaneously limiting phagocytosis, constraining antigen presentation and weakening downstream T‐cell priming [10, 16]. This does not yet establish a melanoma‐specific causal hierarchy, but it does provide a strong rationale for testing CD24‐axis blockade in combination with adaptive immune checkpoint inhibitors, particularly in settings characterized by myeloid enrichment, poor antigen presentation or incomplete responses to PD‐1‐based therapies.

## 6 Clinical Relevance

### 6.1 Prognostic Value

Across multiple solid tumours, cytoplasmic and membranous CD24 expression has been associated with advanced stage, metastatic behaviour and worse clinical outcome [27]. In cutaneous melanoma, the available data point in a broadly similar direction, with CD24 positivity in tumour cells being associated with more aggressive disease features and less favourable outcomes in selected cohorts [7]. However, the melanoma‐specific evidence base remains limited and heterogeneous, and therefore CD24 should not yet be considered a universally validated stand‐alone prognostic biomarker in melanoma.

A more defensible interpretation is that tumour‐cell CD24 marks melanoma states enriched for adverse biologic features, including invasion, stem‐like behaviour, plasticity and therapy resistance [32, 39, 40]. In this framework, CD24 may complement established clinicopathologic and molecular risk stratification approaches rather than replace them. Importantly, prognostic interpretation should always specify the analyte source and assay context, because tissue CD24 measured on melanoma cells is biologically distinct from CD24 detected on immune cells or in circulation [7, 21, 45].

### 6.2 Predictive Value for Immunotherapy

Given its dual association with tumour‐cell plasticity and innate immune suppression, CD24 is an attractive candidate biomarker for predicting response to immunotherapy. Conceptually, tumours with high tumour‐cell CD24 expression and an active CD24–Siglec‐10 axis may rely more heavily on myeloid‐mediated immune suppression and may therefore be less likely to respond to T cell‐focused checkpoint blockade alone [10, 16]. This idea is biologically plausible in melanoma, where effective immunotherapy depends not only on T‐cell activation but also on antigen uptake, phagocytic clearance, dendritic cell function and productive priming of antitumor immunity [53].

At present, however, predictive evidence specific to melanoma remains limited. Most functional support for CD24‐directed therapeutic sensitization derives from broader preclinical cancer literature rather than melanoma‐focused clinical studies [16, 55]. For example, preclinical studies with SWA11, a monoclonal antibody targeting CD24, have shown antiproliferative effects in lung, ovarian and pancreatic cancer cell lines, as well as partial tumour growth control in colorectal models, particularly in combination with cytotoxic agents [25, 57]. Therefore, in melanoma, it is more accurate to describe CD24 as a candidate predictive biomarker and biologically informed enrichment factor rather than an established predictor of response to PD‐1‐ or CTLA‐4‐based therapy. The most promising clinical use case may be to identify patients whose tumours combine CD24‐high tumour states with a Siglec‐10‐positive suppressive myeloid compartment and who may therefore benefit from combinations that pair innate checkpoint blockade with adaptive checkpoint inhibition or targeted therapy [16, 55].

### 6.3 Biomarker Development Across Tissue, Blood and Extracellular Vesicles

CD24 is a versatile biomarker candidate because it can be measured in tumour tissue, peripheral blood and EV [6, 45]. In tissue, CD24 can be assessed by immunohistochemistry, multiplex immunofluorescence, flow cytometry or single‐cell platforms, enabling evaluation of both tumour‐cell expression and its spatial relationship to Siglec‐10‐positive myeloid cells [7, 16]. In blood, CD24 can be measured on leukocyte subsets or in circulating EV, creating minimally invasive opportunities for disease monitoring and pharmacodynamic assessment [21, 47].

At the same time, analytic interpretation requires caution. CD24 is expressed not only by melanoma cells but also by multiple haematopoietic and non‐haematopoietic compartments [21]. Accordingly, circulating or EV‐associated CD24 cannot be assumed to reflect tumour‐cell CD24. Plasma EV populations in patients with melanoma are often dominated by vesicles derived from blood cells, and tumour‐derived EV may require enrichment or multimarker deconvolution for accurate interpretation [45]. This issue is especially important if CD24 is to be developed as a longitudinal biomarker of pathway activity or treatment response.

To address these challenges, biomarker development will need to move beyond single‐epitope readouts and evaluate CD24 in a context‐aware manner. In practice, this means integrating CD24 with multimarker cell‐state panels and orthogonal platforms such as immunohistochemistry, flow cytometry, transcriptomic or proteomic assays and EV profiling, ideally supported by single‐cell and spatial approaches that distinguish tumour‐cell from immune‐cell sources of signal. In parallel, isoform‐ and glycoform‐aware reagents should improve reproducibility and facilitate cross‐study harmonization.

From a translational perspective, composite approaches are therefore likely to be more informative than single‐analyte measurements. Tumour‐cell CD24 may be most useful when interpreted together with myeloid Siglec‐10 status, spatial immune context and broader genomic or transcriptomic predictors of immunotherapy response [16, 58]. Likewise, EV‐associated CD24 may be more informative when considered as part of a broader vesicular signature rather than in isolation [46]. These considerations suggest that the clinical value of CD24 will depend less on binary positivity than on how well CD24‐centred assays capture the relevant tumour–myeloid immunobiology.

### 6.4 Operationalizing ‘CD24‐Axis Activity’ in Melanoma

For translational use, ‘CD24‐axis activity’ should not be inferred from tumour‐cell CD24 expression alone. Rather, we propose that CD24‐axis activity in melanoma be operationalized as the co‐occurrence of: (i) elevated tumour‐cell CD24; (ii) a permissive glycosylation or epitope context consistent with Siglec engagement; and (iii) a Siglec‐10‐positive suppressive myeloid compartment or downstream myeloid inhibitory features [8, 16]. In practice, this could be approximated using tissue‐based assays that quantify tumour‐cell CD24 together with myeloid Siglec‐10 and spatial immune architecture, or with combined tissue‐plus‐liquid‐biopsy approaches that incorporate circulating or EV‐associated CD24 [46, 48].

An analytically robust CD24 biomarker strategy should therefore address at least four variables: specimen type (tissue vs. blood/EV), platform (IHC, multiplex imaging, flow cytometry, single‐cell profiling or EV assays), analyte origin (tumour‐cell vs. immune‐cell CD24) and cutoff definition [53]. Longitudinal interpretation is also likely to matter because CD24 expression can shift with therapy, stress adaptation and cell‐state transitions [39, 40]. These factors argue against viewing CD24 as a static marker and instead support its development as a dynamic, context‐integrated biomarker of melanoma biology and therapeutic vulnerability.

Given the heterogeneity of the CD24 literature, we provide an evidence map grading melanoma‐specificity and strength of support for core claims (Table 1).

**TABLE 1:** Evidence map grading melanoma‐specificity and strength of support for core CD24 claims.

Core claim	Melanoma‐specificity	Perturbation in melanoma	Bottom‐line interpretation	Grade^a^
Therapy‐induced CD24 in BRAFi resistance (SOX2‐linked persister programmes)	Models ✓ (melanoma)	KD/perturbation supports resensitization	Strongest melanoma‐specific support for a contributory (not universal) functional role in therapy tolerance.	A
CD24^+^ stem−/tumour‐initiating enrichment	Models ✓; Patient data limited	Mostly enrichment/sorting; limited KO/rescue	Best interpreted as a state marker with functional enrichment; dependency remains context‐ and assay‐dependent.	B
CD24–Siglec‐10 (human)/Siglec‐G (mouse) innate checkpoint	Cross‐tumour ✓✓; melanoma validation limited	Limited axis‐level perturbation in melanoma	Strong translational rationale, but melanoma‐specific causality and best target node require dedicated testing (humanized systems may be needed).	C
Prognostic association of tumour‐cell CD24	Pt cohorts heterogeneous; cross‐tumour ✓	N/A (biomarker) assay‐dependent	Candidate prognostic marker in selected contexts; requires analytic validity (cell‐of‐origin, platform, cutoffs) to be interpretable.	B/C
Predictive framework: operational ‘CD24‐axis activity’ (tumour CD24‐high + Siglec‐10^+^ myeloid context)	Concept grounded in myeloid biology; melanoma prospective data limited	Not yet clinically validated in melanoma	Useful for research/trial enrichment; should be tested prospectively with harmonized assays and cutoffs.	C/D

Abbreviations: KD, knockdown; KO, knockout; N/A, not applicable.

^a^ Grade key: (A) Melanoma‐specific functional perturbation supports a contributory role; (B) Melanoma enrichment/correlation with limited perturbation; (C) Strong cross‐tumour support with limited melanoma validation; (D) Hypothesis‐generating (requires direct testing). Mixed grades indicate heterogeneity across datasets/assays.

## 7 Therapeutic Targeting of the CD24 Axis

**FIGURE 3:**
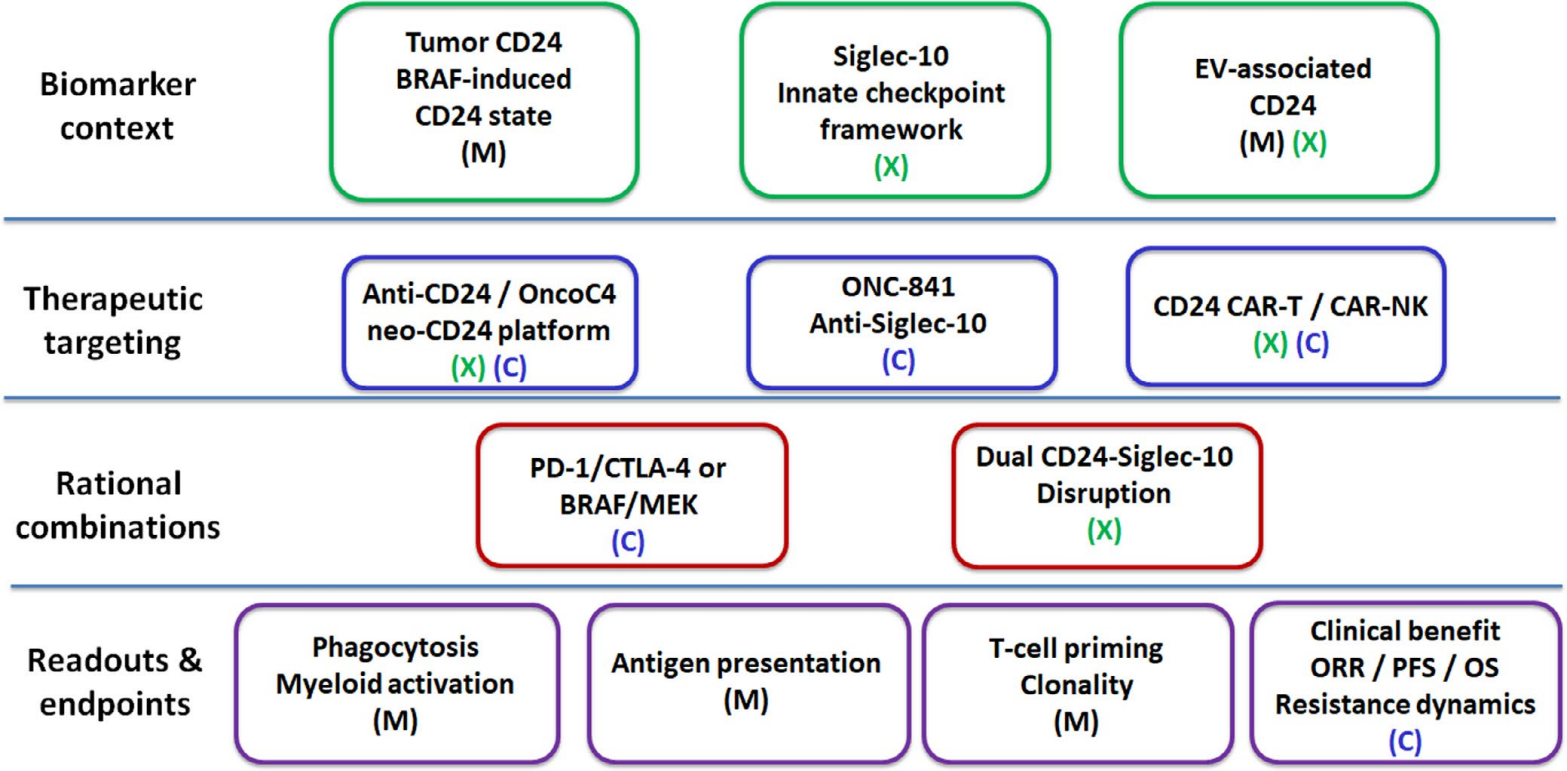
Translational roadmap to inhibit the CD24–Siglec‐10 axis in melanoma. Biomarker‐guided framework linking CD24‐axis biology to therapeutic intervention, rational combinations and mechanistic/clinical endpoints in melanoma. Evidence tags indicate melanoma‐supported (M), extrapolated (X) and clinical/translational (C) elements. The OncoC4 neo‐CD24/ONC‐783 platform is classified as X/C because current support is cross‐tumor/translational rather than melanoma‐specific. Abbreviations: ORR, objective response rate; PFS, progression‐free survival; OS, overall survival.

Because CD24 links tumour‐cell plasticity with innate immune suppression, the CD24 axis is an attractive therapeutic target in melanoma [16, 39]. Current approaches include ligand‐side CD24 blockade, receptor‐side Siglec‐10 inhibition and engineered cellular or multifunctional platforms, most of which remain in preclinical or early translational development [10, 55]. At present, the most defensible therapeutic framework is not to view targeting the CD24 axis as a stand‐alone replacement for established melanoma therapies, but rather as a strategy to reprogramme myeloid immune suppression and thereby complement adaptive checkpoint blockade and, in selected settings, targeted therapy [16, 39]. A proposed translational roadmap linking biomarkers, therapeutic modalities, combinations and readouts is shown in Figure 3. A concise snapshot of the therapeutic landscape is provided in Table 2, and rational combination strategies are summarized in Table 3.

**TABLE 2:** Therapeutic landscape targeting the CD24–Siglec‐10 axis.

Strategy	Agent/platform	Target	Mechanism	Stage	Notes
Direct antibody blockade	Anti‐CD24 monoclonal antibodies (class)	CD24	Disrupts CD24‐axis signalling; may engage Fc‐effector functions (ADCC/ADCP)	Preclinical → early clinical	Most advanced direct approach in the field [55]
Tumour‐selective anti‐CD24	OncoC4 neo‐CD24 platform	Neo‐CD24 (tumour‐associated epitope)	Tumour‐selective recognition of cancer‐associated CD24 glyco‐epitope; adaptable to bispecifics/ADCs/ CARs	Translational/preclinical	Designed to widen the therapeutic window via tumor selectivity [33]. Currently best interpreted as cross‐tumor/translational evidence rather than melanoma‐specific support.
Combination‐rationale mAbs	SWA11	CD24	Proof‐of‐concept anti‐CD24 activity used to motivate combination testing	Preclinical	Reported synergy with cytotoxic therapy in non‐melanoma models [25]
Combination‐rationale mAbs	G7	CD24	Proof‐of‐concept anti‐CD24 activity used to motivate combination testing	Preclinical	Reported combination benefit in non‐melanoma models [55]
Receptor‐side blockade (ICB)	ONC‐841 (anti–Siglec‐10)	Siglec‐10	Blocks the receptor side of the CD24–Siglec‐10 checkpoint to restore myeloid anti‐tumour function	Clinical (first‐in‐human)	Recruiting trial: NCT06352359 [59]
Delivery/PK optimization	Nanoparticle‐based Siglec‐10 blockade (platform)	Siglec‐10	Aims to improve delivery and pharmacokinetics; may reduce systemic exposure	Preclinical/platform	Safety balance is key, given Siglec‐10 on normal immune cells [60, 61]
Adoptive cell therapy	CD24 CAR‐T (e.g., 24BBz; hG7‐BM3)	CD24	CAR‐mediated cytotoxicity against CD24^+^ tumour cells; anti‐tumour activity in xenografts	Preclinical	Supports the feasibility of CD24 as a CAR target [14]
Multi‐antigen adoptive cell therapy	BCMA/CD24 dual CAR‐T	BCMA + CD24	Multi‐antigen targeting to reduce antigen escape; strong tumour control in xenografts	Preclinical	Illustrates multi‐target designs for durability [62]
Innate adoptive cell therapy	CD24 CAR‐NK (platform)	CD24	CAR‐engineered NK cytotoxicity; proposed for synergy with ICB or myeloid modulators	Preclinical/development	Potential ‘off‐the‐shelf’ logistics and lower CRS risk [63, 64]
Clinical anti‐CD24 antibody	IMM47	CD24	CD24‐directed antibody therapy; early‐phase evaluation focused on safety and PK	Clinical (Phase I)	NCT05985083 (primary endpoints: safety, PK) [65]
Clinical fusion protein	CD24‐Fc fusion proteins	CD24 axis	CD24‐based fusion proteins evaluated clinically in immune‐modulatory contexts	Clinical evaluation	Trials referenced: NCT04552704 [66] NCT04060407 [67]

Abbreviations: ADC, antibody–drug conjugate; ADCC, antibody‐dependent cellular cytotoxicity; ADCP, antibody‐dependent cellular phagocytosis; CAR, chimeric antigen receptor; CAR‐NK, CAR‐natural killer cell; CAR‐T, CAR T‐cell; CRS, cytokine release syndrome; ICB, immune checkpoint blockade; mAb, monoclonal antibody; PK, pharmacokinetics.

**TABLE 3:** Rational combinations matrix for CD24‐axis inhibition in melanoma.

Backbone (CD24‐axis)	Partner	Mechanistic rationale	Selection biomarkers	Endpoints/readouts
Anti‐CD24 or anti–Siglec‐10	PD‐1/PD‐L1 blockade	Layered checkpoint targeting: restores myeloid clearance/antigen presentation while reinvigorating exhausted T cells [15, 56]	Tumour CD24 (IHC/flow); Siglec‐10high TAMs; PD‐L1; T‐cell–inflamed signature	ORR/PFS; T‐cell activation/infiltration; myeloid activation; phagocytosis assays; cytokines
Anti‐CD24 or anti–Siglec‐10	CTLA‐4 blockade	Combines innate checkpoint relief with enhanced priming/expansion of anti‐tumour T cells [5, 16]	CD24/Siglec‐10 axis activity; low baseline priming/DC activation; intratumoral Treg abundance	Tumour control; priming metrics; clonality; Treg/Tconv balance; immune‐related toxicity
Dual CD24–Siglec‐10 disruption (e.g., engineered scaffolds)	PD‐1/PD‐L1 blockade	Preclinical synergy reported, including reversal of PD‐1 resistance when both axes are targeted [56]	PD‐1‐refractory disease; elevated CD24/Siglec‐10 signalling; myeloid‐dominant TME	Resensitization to PD‐1; durability; serial immune profiling; pathway engagement biomarkers
Anti‐CD24 or anti–Siglec‐10	BRAF/MEK inhibitors (MAPK pathway)	MAPK inhibition can induce adaptive drug‐tolerant states; CD24 induction via SOX2/STAT3 signalling supports combination therapy [39]	BRAF V600; on‐treatment CD24 induction; SOX2/STAT3 activity (if available)	Time‐to‐resistance; MRD/relapse delay; scheduling effects; longitudinal CD24 dynamics
Anti‐CD24	Chemotherapy and/or radiotherapy	ICD/antigen release plus improved myeloid‐mediated clearance and immune activation; supported by combination rationale [25, 56]	CD24‐high tumours; immune‐cold baseline; impaired phagocytosis signatures; ICD markers (if assessed)	Local/systemic control; DC activation; immune infiltration; tolerability; circulating biomarkers
Anti‐CD24 or anti–Siglec‐10	Other innate checkpoints (e.g., CD47–SIRPα)	Mitigates compensatory ‘don't‐eat‐me’ signalling and may deepen/extend myeloid reprogramming [16, 54, 60]	Co‐expression of CD24 and other innate checkpoints; high TAMs/MDSCs infiltration; low T‐cell infiltration	Phagocytosis; TAM polarization; durability of response; safety/toxicity profiling

Abbreviations: CTLA‐4, cytotoxic T‐lymphocyte‐associated protein 4; DC, dendritic cell; ICD, immunogenic cell death; IHC, immunohistochemistry; MAPK, mitogen‐activated protein kinase; MDSCs, myeloid‐derived suppressor cells; MEK, MAPK/ERK kinase; MRD, minimal residual disease; ORR, objective response rate; PD‐1, programmed cell death protein 1; PD‐L1, programmed death‐ligand 1; PFS, progression‐free survival; TAMs, tumour‐associated macrophages; Tconv, conventional CD4 T cells; TME, tumour microenvironment; Treg, regulatory T cells.

### 7.1 Direct Anti‐CD24 Antibodies

Direct targeting of CD24 with monoclonal antibodies is the most developed ligand‐side strategy and has shown broad preclinical activity across solid tumours [55, 57]. In principle, anti‐CD24 antibodies may act through several non‐mutually exclusive mechanisms: blockade of CD24–Siglec‐10 engagement, interference with CD24‐associated tumour‐cell programmes and Fc‐dependent effector functions such as antibody‐dependent cellular phagocytosis (ADCP) and antibody‐dependent cellular cytotoxicity (ADCC) [16, 25, 55]. This multimodal potential is attractive in melanoma, where tumour‐cell plasticity and myeloid suppression may coexist within the same lesion. Early proof‐of‐concept antibodies (e.g., SWA11 and G7) illustrate feasibility for direct CD24 engagement and provide precedent for Fc‐enabled activity in preclinical systems [25, 55, 57].

A key translational opportunity is the development of tumour‐selective anti‐CD24 formats. Because CD24 is heavily glycosylated and may display cancer‐associated glyco‐epitopes, engineered antibodies recognizing aberrantly glycosylated or tumour‐restricted CD24 forms could widen the therapeutic window relative to non‐selective CD24 blockade [19, 33]. OncoC4 has described a cancer‐selective neo‐CD24 platform that recognizes a tumour‐associated neo‐CD24 epitope exposed on malignant cells. Public materials indicate that this platform includes ONC‐783 and is conceived as a modular strategy that may be adaptable across multiple therapeutic formats, rather than as ONC‐841, which targets Siglec‐10. Because current public information describes ONC‐783/neo‐CD24 as a CD24‐directed oncology platform at a preclinical/IND‐enabling stage rather than as a melanoma‐specific program, we classify this approach as cross‐tumor/translational evidence (X/C), not melanoma‐supported evidence (M). Such approaches may be particularly relevant in melanoma, where improving tumour selectivity while preserving myeloid reprogramming potential could strengthen the risk–benefit profile. However, most efficacy data for direct anti‐CD24 antibodies derive from non‐melanoma systems [33, 55]. Accordingly, in melanoma, these agents should currently be viewed as plausible translational candidates rather than clinically validated therapies.

### 7.2 Anti–Siglec‐10 Strategies

Receptor‐side blockade provides a complementary approach to disrupt the axis and may be particularly attractive when tumour‐cell CD24 expression is heterogeneous, dynamic or distributed between tumour cells and EV [10, 39, 45]. By targeting Siglec‐10 on suppressive myeloid cells, this strategy aims to restore phagocytosis, antigen uptake and inflammatory activation even when the ligand source is multifocal or evolving over time [10, 16]. Conceptually, this may be advantageous in melanoma, where cell‐state transitions and therapy‐induced CD24 upregulation could complicate purely ligand‐centred approaches [39, 40].

At the same time, receptor‐side targeting raises distinct challenges. Siglec‐10 is expressed on normal immune compartments, so systemic blockade may carry a greater risk of broad myeloid disinhibition or inflammatory toxicity than tumour‐restricted ligand‐side approaches [10, 60]. For this reason, delivery approaches that concentrate activity in tumours or lymphoid tissues, including nanoparticle‐based strategies and other engineered platforms, may be especially relevant for Siglec‐10‐directed therapy [61]. At present, neither ligand‐side nor receptor‐side targeting can be considered clearly superior in melanoma. Anti‐CD24 may offer better selectivity when tumour‐specific epitopes are exploitable, whereas anti–Siglec‐10 may be attractive when pathway activity is driven by heterogeneous or non‐cell‐autonomous CD24 sources [10, 60]. Notably, ONC‐841 (anti–Siglec‐10) has entered first‐in‐human testing in advanced solid tumours (NCT06352359) [59], underscoring the translational momentum for receptor‐side blockade. Early clinical development of anti–Siglec‐10 agents supports the translational relevance of receptor‐side blockade, although melanoma‐specific efficacy remains to be established.

### 7.3 Cell‐Based and Engineered CD24‐Directed Modalities

Cell‐based and multifunctional engineered approaches further expand the therapeutic landscape of the CD24 axis. Cell‐based strategies extend CD24 targeting into CAR platforms; for example, CD24‐directed CAR‐T designs such as 24BBz CAR‐T incorporating a humanized CD24 scFv (hG7‐BM3) have been reported to reduce tumour growth and metastatic burden in preclinical models [14]. In parallel, multi‐antigen designs (e.g., BCMA/CD24 dual CAR‐T) have been proposed as a pragmatic strategy to mitigate antigen escape and broaden target coverage [62]. CD24‐directed CAR‐NK platforms are also emerging and may offer practical or safety advantages, including the possibility of ‘off‐the‐shelf’ use and a potentially lower risk of some toxicities associated with autologous CAR‐T approaches [63, 64]. Although these platforms have not yet been established in melanoma, the biology summarized in earlier sections supports their consideration in selected high‐risk or biomarker‐enriched settings.

Engineered dual‐targeting or multifunctional formats may be particularly appealing in melanoma because they can address multiple barriers simultaneously. For example, constructs that simultaneously disrupt CD24/Siglec‐10 signalling and adaptive checkpoint pathways, or that combine CD24 targeting with additional tumour‐ or microenvironment‐specificities, may increase potency while improving selectivity [56, 68]. In this sense, the CD24 field may ultimately advance more through platform matching than through a single universal modality, in which the optimal intervention is chosen based on tumour‐cell CD24 biology, myeloid context and therapeutic setting.

### 7.4 Rational Combination Strategies

The strongest rationale for CD24‐axis inhibition in melanoma lies in its potential for combination therapy. Because the pathway links innate immune suppression with therapy‐tolerant tumour states, CD24‐axis targeting is best positioned as an innate‐checkpoint‐directed partner layered onto currently used melanoma regimens rather than as a monotherapy backbone [16, 39]. Mechanistically, restoring macrophage phagocytosis and antigen handling could complement PD‐1‐based therapy by improving antigen presentation and downstream T‐cell priming [16, 56].

This logic supports several combination settings. First, CD24‐axis inhibition may complement PD‐1 monotherapy in tumours with incomplete antigen presentation or dominant myeloid suppression. Second, it may further enhance dual‐checkpoint regimens, including PD‐1 plus CTLA‐4 and potentially PD‐1 plus LAG‐3, by addressing an innate barrier not directly targeted by adaptive checkpoint blockade [16, 56]. Third, in BRAF‐mutant melanoma, combining CD24‐axis inhibition with BRAF/MEK‐targeted therapy is mechanistically attractive because MAPK‐targeted treatment can induce SOX2‐ and CD24‐linked adaptive states associated with therapy resistance [39]. In this context, CD24‐axis inhibition could potentially blunt the emergence of drug‐resistant persister‐like populations while also improving innate immune engagement [16, 39].

Preclinical support for this combinatorial concept already exists. Dual‐targeting scaffolds that simultaneously disrupt CD24/Siglec‐10 and PD‐1/PD‐L1 interactions have shown synergistic antitumor effects, including in melanoma models [56]. More broadly, these findings support a translational roadmap in which CD24‐axis inhibition is paired with adaptive checkpoint blockade, MAPK‐targeted therapy or other immune‐activating modalities, based on tumour genotype, immune contexture and biomarker evidence of CD24‐axis activity [39, 40, 56].

### 7.5 Therapeutic Feasibility, Safety and Risk Mitigation

An important clinical question is not only whether the CD24 axis is targetable, but also how to target it safely. CD24 is expressed beyond tumour cells, including on haematopoietic and other normal cell populations, while Siglec‐10 is present on normal immune compartments [10, 21]. As a result, both ligand‐side and receptor‐side interventions raise on‐target/off‐tumour considerations. For anti‐CD24 approaches, potential concerns include unintended engagement of normal CD24‐positive cells and excessive Fc‐mediated depletion or activation. For anti–Siglec‐10 strategies, the principal concern is broader myeloid disinhibition, with the potential for unwanted inflammatory toxicity [55, 60].

These issues make modality design especially important. Tumour‐selective glyco‐epitope recognition, conditional activation systems, bispecific targeting formats and Fc engineering may all help widen the therapeutic window [33, 68]. Likewise, tumour‐directed or compartment‐restricted delivery strategies may reduce systemic exposure for Siglec‐10 blockade [61]. For cellular therapies, combinatorial antigen logic and careful antigen‐density thresholds may be necessary to improve selectivity [62, 64]. At present, the field does not support a definitive conclusion that blocking CD24 is more clinically relevant than blocking Siglec‐10 in melanoma. Rather, the choice may ultimately depend on whether the dominant vulnerability in each tumour is ligand abundance, receptor‐positive myeloid dependency or a broader state of CD24‐axis activity involving both.

### 7.6 Clinical Development and Biomarker‐Guided Translation

Clinical translation of the CD24 axis is underway, with early‐phase studies involving anti‐CD24 antibodies, CD24‐Fc–based approaches and anti–Siglec‐10 therapies [10, 59, 65, 66]. Notably, a melanoma‐specific CD24Fc trial combining CD24Fc with ipilimumab and nivolumab to reduce immune‐related adverse events was registered as NCT04060407 but was withdrawn before enrollment; therefore, it should be interpreted as evidence of clinical interest in this axis rather than evidence of melanoma efficacy [67]. Although early clinical evaluation is ongoing, melanoma‐specific efficacy signals remain undefined. This makes biomarker‐guided development especially important. The most informative strategy is unlikely to rely solely on tumour CD24, but rather on an integrated assessment of tumour‐cell CD24 abundance, glycosylation or epitope context, myeloid Siglec‐10 expression, spatial immune architecture and circulating or EV‐associated CD24 [10, 45, 48].

Accordingly, early‐phase melanoma studies should incorporate mechanism‐linked endpoints in addition to conventional efficacy measures. These could include macrophage phagocytosis, antigen uptake, myeloid‐cell reprogramming, T‐cell infiltration and activation and longitudinal blood‐based biomarkers of pathway activity [16, 48, 52]. Such biomarker‐rich designs will be essential for determining patient selection, therapeutic sequencing and the most appropriate modality for CD24‐axis intervention in melanoma.

### 7.7 Major Open Questions

Several important questions remain. First, what is the optimal therapeutic modality for melanoma: direct anti‐CD24 blockade, anti–Siglec‐10 targeting, engineered dual‐function constructs or cellular therapy? Second, which combination partners and treatment sequences are most effective, particularly in relation to PD‐1‐based immunotherapy, dual‐checkpoint regimens and BRAF/MEK‐targeted therapy? Third, how does CD24‐axis activity interact with other resistance programmes in melanoma, including defective antigen presentation, interferon‐pathway dysfunction, immune exclusion and state plasticity?

Addressing these questions will require melanoma‐focused studies that integrate tissue‐based and liquid‐biopsy biomarkers with functional immune readouts. If successful, such work could establish the CD24 axis not only as a biomarker of aggressive disease states but also as a modifiable therapeutic vulnerability linking tumour‐cell plasticity to innate immune evasion.

Additional mechanistic priorities include:

- Inflammasome: Determine whether CD24‐associated melanoma states co‐segregate with inflammasome activity (e.g., IL‐1β/IL‐18 signatures) and whether CD24‐axis perturbation alters inflammasome outputs in tumour–myeloid co‐cultures and in vivo [69].
- Internalization and surface dynamics: Define whether CD24 undergoes ligand‐ or stress‐dependent redistribution or internalization in melanoma and whether functional effects track with surface versus intracellular pools (e.g., antibody‐feeding assays, live imaging, surface biotinylation) [31].
- Lineage‐state context (MITF axis) and genotype: Test whether CD24 tracks with MITF‐high proliferative versus MITF‐low/dedifferentiated trajectories, and whether common melanoma genotypes (e.g., CDKN2A loss) modify CD24‐associated phenotypes using genotype‐stratified perturbation and single‐cell or spatial analyses [40, 43].
- CD133^+^/CD24^+^ compartments and phenotype switching: Assess overlap and stability of CD133 and CD24 states under therapy pressure, including slow‐cycling persister programmes described in melanoma [70, 71].
- Melanoma‐specific loss‐of‐function: Prioritize CRISPR knockout and rescue experiments to distinguish association from requirement across growth, invasion, persister formation and therapy resistance [32, 39].
- Molecular organization: Because CD24 is GPI‐anchored, evaluate whether cholesterol‐dependent clustering or raft partitioning (monomeric vs. higher‐order assemblies) governs co‐receptor scaffolding and phenotype outputs in melanoma [26].
- Axis crosstalk: Dissect whether Siglec‐10 blockade alters ‘tumour‐intrinsic’ CD24‐associated programmes indirectly through immune pressure, rather than through direct intracellular signalling, by comparing tumour‐only with tumour–myeloid co‐culture systems [16].

## 8 Conclusions and Perspectives

At present, CD24 is best viewed not as a universally established melanoma dependency, but as a context‐dependent marker and potential mediator of aggressive tumour‐cell states and innate immune suppression [16, 32, 39]. The available literature supports a framework in which CD24‐enriched melanoma states may intersect with stem‐like behaviour, therapy resistance, stress adaptation and impaired myeloid antitumor function, while also highlighting substantial gaps in melanoma‐specific functional validation [16, 32, 39, 49]. Several important mechanistic concepts in this field remain supported more strongly by broader cancer or immunology literature than by melanoma‐focused perturbation studies [10, 25].

This distinction has practical implications for both biomarker development and therapeutic translation. Clinically, the most promising path forward is unlikely to rely on CD24 positivity alone, but rather on integrated assessment of tumour‐cell CD24, glycosylation or epitope context, myeloid Siglec‐10 status, spatial immune architecture and circulating or EV‐associated CD24 [16, 45, 48]. Therapeutically, CD24‐axis intervention is best positioned as a biomarker‐guided strategy to complement established melanoma treatments, particularly adaptive immune checkpoint blockade and, in selected settings, MAPK‐targeted therapy [16, 39, 40].

Several questions now define the next phase of the field: which component of the axis should be targeted in melanoma; which modality offers the best balance of efficacy and safety; how CD24‐axis activity interacts with other resistance programmes; and which biomarker framework most accurately identifies pathway‐dependent tumours [10, 16, 48]. Addressing these questions will require melanoma‐focused studies that combine tissue‐based and liquid‐biopsy biomarkers with functional immune readouts and careful therapeutic stratification. If successful, such work could establish CD24 not only as a marker of adverse melanoma states but also as a clinically actionable vulnerability linking tumour‐cell plasticity to innate immune evasion [16, 39].

## Author Contributions

Conceptualization, A.P.M.; Writing original draft preparation, C.L., R.C.C. and N.C.N.; Writing review and editing, Y.W, A.G., K.T.A. and A.P.M. All authors have read and agreed to the published version of the manuscript.

## Funding

This research received no external funding. K.T.A. was funded by Swim Across America.

## Conflicts of Interest

The authors declare no conflicts of interest.

## Data Availability

Data sharing not applicable to this article as no datasets were generated or analysed during the current study.
